# RNA-Seq Reveals the Underlying Molecular Mechanism of First Cleavage Time Affecting Porcine Embryo Development

**DOI:** 10.3390/genes13071251

**Published:** 2022-07-15

**Authors:** Xinhui Song, Tiantian Li, Xin Xiong, Huiquan Shan, Tong Feng, Kuiqing Cui, Deshun Shi, Qingyou Liu, Zhipeng Li

**Affiliations:** 1 State Key Laboratory for Conservation and Utilization of Subtropical Agro-Bioresources, Guangxi University, Nanning 530004, China; songxinhui@st.gxu.edu.cn (X.S.); huiquan_shan@163.com (H.S.); fengtong_bio@163.com (T.F.); kqcui@gxu.edu.cn (K.C.); ardsshi@gxu.edu.cn (D.S.); 2 Guangdong-Hong Kong-Macao Central Nervous Research Institute, Medical School, Jinan University, Guangzhou 510632, China; ltt525212022@163.com (T.L.); xiongx0505@jnu.edu.cn (X.X.); 3 Guangdong Provincial Key Laboratory of Animal Molecular Design and Precise Breeding, School of Life Science and Engineering, Foshan University, Foshan 528225, China; qyliu-gene@gxu.edu.cn

**Keywords:** RNA-seq, porcine, embryo evaluate, parthenogenetic activation, IVF

## Abstract

The selection and evaluation of high-quality embryos are the key factors affecting in vitro embryo development and pregnancy outcome. The timing of first embryonic cleavage has been considered a positive indicator of the in vitro developmental potential of embryos, while the underlying molecular mechanism is still not fully understood. In this study, the embryos generated by parthenogenetic activation (PA) or in vitro fertilization (IVF) were monitored and recorded every 2 h and divided into two groups (early cleavage or late cleavage) based on the cleavage rate and blastocyst formation data. RNA sequencing was used to analyze the gene expression pattern of the embryos. We identified 667 and 71 different expression genes (DEGs) in early cleavage and late cleavage porcine PA and IVF embryos, respectively. Further Gene Ontology (GO) and Kyoto Encyclopedia of Genes and Genomes (KEGG) analyses showed that the DEGs are mainly enriched in pathways concerning the proteasome, DNA repair, cell cycle arrest, autophagy, and apoptosis, suggesting that severe endoplasmic reticulum stress (ERS) and DNA damage may be the key factors that led to the low development potential of late cleavage embryos. This study provides a theoretical basis for the following application and offers important information about the understanding of the timely manner of porcine embryo development.

## 1. Introduction

Though embryo production in vitro has been widely studied and used in various fields, embryo quality and pregnancy rates are still at a relatively low level. These lower pregnancy rates may be caused, in part, by the negative impact of in vitro culture on the oocytes. The nonhomogeneous development of the oocytes may also affect the pregnancy rate [1]. Furthermore, simply surviving the experience of pre-implantation does not mean it can develop into a normal embryo, fetus, neonate, and adult. Multiple methods have been proposed to evaluate embryo quality during in vitro culture, and the standard morphologic evaluation is the most commonly used non-invasive method [2]. However, the outer appearance might not always reflect the embryo’s developmental potential [3,4,5]. Therefore, the selection of in vitro cultured embryos remains a subjective process in most mammalian species, and exploration of the precise way to identify optimal embryos before implantation is of major importance.

Recently, the timing of the first cleavage was used as a strong marker of embryonic viability and represents an ideal method of embryo selection in rapid, simple, accurate, and noninvasive ways. Studies have found that embryos produced in vitro commonly show heterogeneity in their development timing concomitant with variability in developmental competence [6]. Those embryos that develop on time have the highest developmental competence and viability after a transfer, while what the timely manner is remains the key question. Relatively early cleavage embryos are usually more likely to develop into high-quality blastocysts [7,8], while embryos cleaving too early or late are also indicative of poor embryo quality [9,10]. Diverse studies have found that relatively early cleavage embryos relation to better developmental competence than those cleaving later embryos in pig [11,12], human [13], buffalo [14], cattle [7,15], goat [16], hamster [17], and mouse [18]. It has been reported that early cleavage embryos exhibited higher expression in genes that participated in the meiotic cell cycle, while enrichment of RNA processing- and translation-related genes was found in porcine late cleavage embryos of somatic cell nuclear transfer (SCNT) [19]. While the delay of cell division might be a consequence of chromosomal aberrations and DNA damage [20], slow cleaving embryos have a higher caspase activity in comparison to fast cleavers [21]. However, the gene expression pattern which may uncover the timely manner underlying embryo development has still not been fully revealed.

Here, using the Smart-seq2 technology, we analyzed the gene expression pattern of early cleavage and late cleavage porcine in vitro cultured 2-cell embryos and investigated the relationship between the cleavage time and developmental potential of porcine embryos. This study provides important information about the understanding of the timely manner of porcine embryo development and reveals the underlying molecular mechanism of first cleavage time affecting embryo development.

## 2. Materials and Methods

### 2.1. Collection of Porcine Oocytes

About 200 ovaries of landrace pigs (about 8–10 months) were used in this study. The ovaries were obtained from a local abattoir. Referring to a previous report [22], the ovaries were transported to the laboratory in 0.9% (*w*/*v*) NaCl sterile solution at 33 °C within 2 h. Cumulus–oocyte-complexes (COCs) were aspirated from 3 to 6 mm follicles with a 10 mL syringe and washed with cell culture medium (CCM) containing TCM 199 (GIBCO), supplemented with 2% (*v*/*v*) FBS (GIBCO), 0.9 μg/mL NaCl, 1.2 μg/mL HEPES and 0.4 μg/mL NaHCO_3_.

### 2.2. In Vitro Maturation (IVM) of Porcine Oocytes

According to a previous report [22], COCs with uniform cytoplasm and at least three layers of compact cumulus cells were selected under an inverted optical microscope and were placed in the basic maturation medium (MM) consisting of TCM 199 with the addition of 0.6 mM cysteine, 10 ng/mL IGF-I, 50 ng/mL EGF, and 10% (*v*/*v*) porcine follicular fluid. COCs were matured in MMH (MM supplemented with 15 IU/mL ECG and 10 IU/mL hCG) during the first 22 h and then cultured in MM without hormone for another 20 h at 38.5 °C under humidified air containing 5% CO_2_.

### 2.3. Parthenogenetic Activation (PA)

After IVM, COCs were denuded mechanically by repeated pipetting in 0.1% hyaluronidase for removing cumulus cells (CCs). Only matured oocytes with an extruded first polar body and uniform cytoplasm were used for further experiments. Thirty oocytes were washed three times with electrical activation medium containing 0.3 mM mannitol, 0.1 mM CaCl_2_, 0.1 mM MgSO_4_, 0.5 mM HEPES, and 0.01 g/L BSA and then transferred into a BTX (BTX, San Diego, CA, USA) covered with electrical activation medium. Electrical stimulation was performed referring to a previous study [23]. Three direct current (DC) pulses of 50 V/cm for 80 µsec were applied to oocytes with a Cell Fusion-Active device (BLS CF-150/B). Then, every 5–8 activated embryos were cultured for 7 days in a 35 µL droplet of modified PZM-3 medium (containing 108 mM NaCl, 10 mM KCl, 0.35 mM KH_2_PO_4_, 7 mM NaHCO_3_). These culture droplets were covered with mineral oil and incubated at 38.5 °C in 5% CO_2_ and maximum humidity in advance.

### 2.4. In Vitro Fertilization (IVF)

Fresh semen of Duroc pig was obtained from the local livestock breed improvement station. IVF was performed according to a previous reference [24] with slight modifications; 0.8 mL semen was diluted with 6 mL semen diluent solution mTBM (containing 111.3 mmol/L NaCl, 5.0 mmol/L Sodium pyruvate, 3.0 mmol/L KCl, 7.5 mmol/L CaCl_2_, 11.0 mmol/L Glucose, 0.2% BSA, 20.2 mmol/L Tris) and centrifuged with 1200× *g* for 5 min at room temperature, and 0.5 mL semen was saved and transferred into 5 mL fertilization fluid (PF) containing mTBM and 2 mmol/L Caffeine and centrifuged with 1200× *g* for 3 min. A volume of 0.3 mL semen was saved and transferred into a 1.5 mL EP tube for IVF. A volume of 500 μL of sperm suspension was added to 1 mL of culture medium containing oocytes, and the final sperm concentration was 2.0 × 10^6^ cells/mL. Every 25 matured oocytes surrounded with sperm were co-cultured in PF medium for 2 h. Fertilized embryos were cultured in PZM-3 medium for 7 days.

### 2.5. Cultivation and Statistics of Embryos Cleaved at Different Time

During 12–48 h after activation and fertilization, the growth and cleavage of embryos were observed every two hours. Once the embryos came up to the 2-cell stage, they were picked out to a new plate and continued the culture. The time and quantity were marked clearly and recorded. All the blastocysts were observed on the 7th day. Embryo development and quality among different periods were observed and statistically analyzed. Blastocysts at different periods were counted and collected. The cleavage rate of embryos cleaved at different times as well as the blastocyst rate were calculated.

### 2.6. Assessments of Nucleus Number in Blastocysts of PA Embryos

Collection of blastocysts was accomplished on the 7th day after activation. Blastocysts were classified into four levels, namely the primary blastocyst, initial blastocyst, expanded blastocyst, and hatched or hatching blastocyst. Blastocysts were fixed with 4% paraformaldehyde in a DPBS (Dulbecco’s Phosphate Buffered Saline) for 10 min at room temperature. After fixation, samples were washed with PBS containing 1 mg/mL polyvinyl alcohol (PBS-PVA) and stained with 10 μg/mL Hoechst 33,342 for 15–20 min [23]. Thereafter, blastocysts were rinsed with PBS-PVA, mounted on a glass slide, and gently flattened with coverslips. The stained blastocysts were evaluated using a fluorescence microscope. The number of nuclei in each blastocyst was counted. A digital image of each embryo was taken, and cell numbers were counted using the Image J software (National Institutes of Health, Bethesda, MD, USA).

### 2.7. RNA Sequencing of Embryos

PA 2-cell embryos were divided into two groups with one repeat in each group, while IVF 2-cell embryos were divided into two groups with two repeats in each group. The embryos were collected in the cell lysis buffer with 20–30 embryos per pool, and Oligo dT was used for reverse transcription to the 1st cDNA. PCR was performed to enrich the nucleic acid, and the products were purified to construct the library, including DNA fragmentation, end-repair, poly-A adaptation, PCR amplification, library quality control, etc. The libraries were sequenced by an Illumina Hiseq platform with 150 bp paired-end. The original off-machine sequence (raw reads) obtained from HiSeq sequencing is processed to obtain high-quality sequences (clean reads) through the processes of removing low-quality sequences and removing connector contamination. All subsequent analyses are based on clean reads. The clean reads were mapped onto the pig reference genome (Sus_scrofa 11.1) by the HISAT2 software (http://ccb.jhu.edu/software/hisat2 (accessed on 22 September 2021) or http://github.com/infphilo/hisat2 (accessed on 22 September 2021), version 2.0.1 or later) [25]. After assessing sequence saturation and read distribution on the reference genome, transcripts of each sample were assembled by StringTie (v1.3.2), and FPKM (fragments per kilobase of transcript sequence per million base pairs sequenced) of each gene was calculated based on the read count file obtained by HTSeq (v0.6.0). The differentially expressed genes were strictly screened using DESeq2 (v1.18.0) with |log_2_ Fold Change| ≥ 1 and FDR (false discovery rate) < 0.05 as parameters, and analyzed using GO (Gene Ontology; http://www.geneontology.org/; accessed on 22 September 2021) and KEGG (Kyoto Encyclopedia of Genes and Genomes; http://www.kegg.jp/; accessed on 22 September 2021). GO and KEGG parameter settings both used a *p*-value < 0.05.

### 2.8. Quantitative Real-Time PCR

cDNA was synthesized by reverse transcription of RNA extracted from early cleavage embryos and late cleavage embryos [23]. Each 20 μL reaction contained 1 μL cDNA, 1 μL of 10 μmol/L forward and reverse primers, 10 μL FastStart Universal SYBR Green Master (Roche, Basel, Switzerland), and 8 μL nuclease-free water. The amplification consisted of an initial denaturation step at 95 °C for 5 min, followed by 40 cycles of denaturation at 95 °C for 15 s, annealing at 60 °C for 30 s, and extension at 72 °C for 1 min. The primer sequences used in this study are listed in Tables S1 and S2. Relative gene expression levels were calculated using the 2^−ΔΔCT^ method with 18S as the housekeeping gene.

### 2.9. Statistical Analysis

All experiments were repeated three times, and SPSS version 17.0 was used for data analyses. Differences between the groups were analyzed with a *t* test. Values are expressed as the mean ± SEM, and a value of *p* < 0.05 was used to indicate statistical significance.

## 3. Results

### 3.1. Relationship between the Timing of First Cleavage and Embryo Developmental Potential of PA

**Figure 1:**
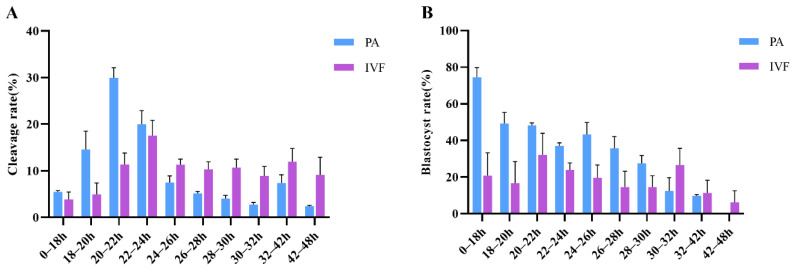
Cleavage and blastocyst rate of embryos cleaved at different times after parthenogenetic activation (PA) or in vitro fertilization (IVF) with four repetitions. (**A**) Cleavage rate of porcine PA and IVF embryos. (**B**) Blastocyst rate of porcine PA and IVF embryos.

The cleavage and blastocyst formation of embryos generated by PA were monitored and recorded every two hours (Table 1). Results showed that the oocytes cleaved at 20–22 h after PA presented the highest cleavage rate, and most of the blastocysts were formed in the early cleaved embryos (Figure 1A,B). Based on the cleavage rate and blastocyst formation data, the embryos were divided into two groups. Embryos that completed the first cleavage in less than 22 h were defined as the early cleavage group (EC group), and those in more than 26 h were defined as the late cleavage group (LC group). Through data analysis, the blastocyst rate of the PA embryos in the EC group is significantly higher than that of the LC group (51.2 ± 2.2% vs. 27.2 ± 6.0%; Table 2). Similar results were observed in the cleavage rate (Table 3). We further analyzed the blastocyst cell number of the PA embryos and found that the cell number of blastocysts from the EC group is also significantly higher than the LC group (44.5 ± 4.2% vs. 33.45 ± 2.7%; 49.7 ± 4.1% vs. 40.26 ± 3.3%; 74.8 ± 2.09% vs. 50.74 ± 3.7%; 65 ± 5.6% vs. 0; Table 2). These results indicated that early cleavage PA embryos have better developmental competence.

### 3.2. First Cleavage Time and Developmental Competence of IVF Embryos

For the IVF embryos a similar analysis was performed (Table 3), and they were also divided into two groups, zygotes that completed the first cleavage in less than 24 h (EC group) or more than 30 h (LC group). Comprehensive data analysis showed that the blastocyst rate of the IVF embryos in the EC group is significantly higher than that of the LC group (28.9 ± 3.9% vs. 10.7 ± 1.9%; Table 2). These results indicated that the developmental potential of early cleavage embryos is significantly better than the later cleavage embryos.

### 3.3. RNA-Seq of the PA Embryos

**Figure 2:**
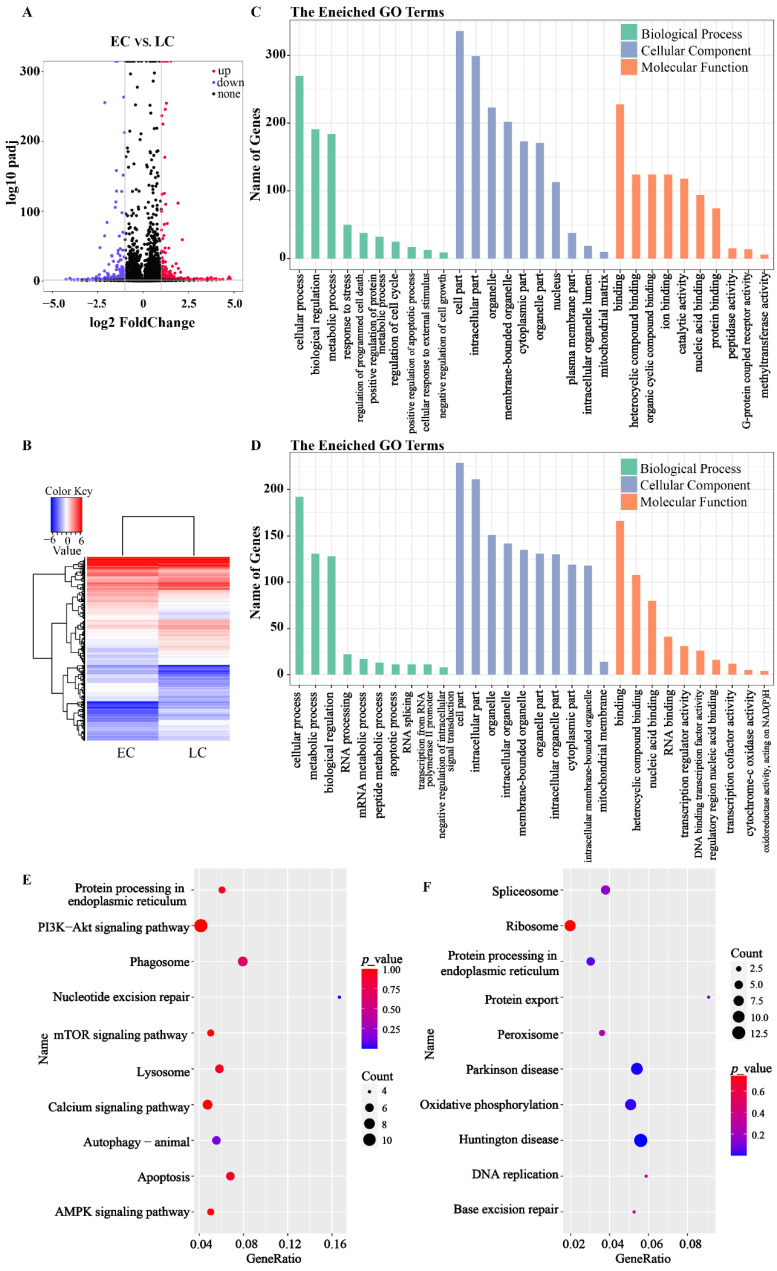
DEGs analysis between pig early cleavage embryos and late cleavage embryos of PA with one repeat per group. (**A**) The volcano plot shows the upregulation and downregulation of DEGs between early and late cleavage pig PA 2-cell embryos (|log_2_ FC| ≥ 1, FDR < 0.05); The red dots represent upregulated genes, and the blue dots represent downregulated genes. (**B**) Heatmaps of DEGs in early and late cleavage pig PA 2-cell embryos. GO enrichment analysis of (**C**) upregulated and (**D**) downregulated DEGs. KEGG pathway enrichment analysis of (**E**) upregulated and (**F**) downregulated DEGs.

**Figure 3:**
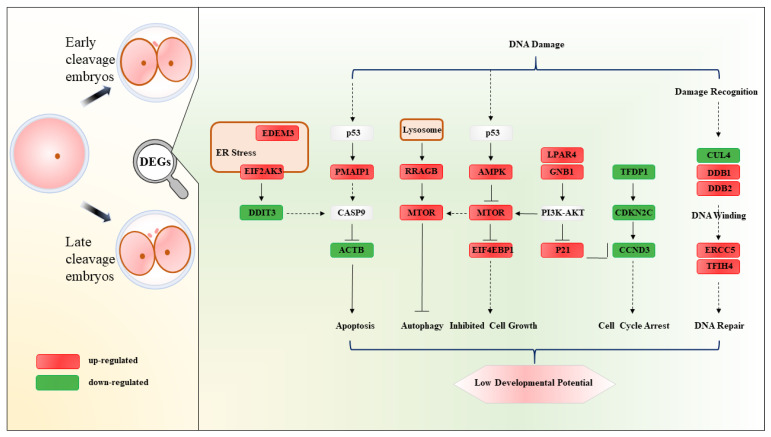
Schematic diagram of pathways by which ER stress- and apoptosis-related genes cause the cleavage postponement of embryos. Genes in red or green indicate significant high expression in LC or EC embryos, respectively.

RNA-seq was performed to analyze the gene expression pattern underlying the early cleavage and late cleavage embryos. In total, 667 different expression genes (DEGs; |log_2_FC| ≥ 1, FDR < 0.05), including 274 downregulated genes and 393 upregulated genes, were found in PA embryos (Figure 2A,B). Further GO analysis found that the DEGs were significantly enriched in the cellular components and molecular function term (Table S3), indicating that the DEGs were mainly involved in the organelle-related activity. The DEGs relation to development and apoptosis were mainly enriched to 30 GO terms, including the mRNA metabolic process, cytochrome-c oxidase activity, and oxidoreductase activity (Figure 2C,D). Further KEGG analysis found that all DEGs were enriched in 275 pathways, and multiple important pathways were found to disadvantage embryo development (Figure 2E,F), including nucleotide excision repair (NER), apoptosis, and autophagy. The expression of genes concerning NER was significantly higher in the late cleavage embryos, suggesting that DNA damage may be one of the main factors adverse to the development of late cleavage embryos. Moreover, the DEGs were also found enriched in the lysosome, phagosome, spliceosome, and protein processing in the endoplasmic reticulum (Figure 2E,F). The gene expression levels of *EDEM3* and *EIF2AK3* in late cleavage two-cell embryos were significantly higher than that in early cleavage embryos (Figure 3).

### 3.4. RNA-Seq of the IVF Embryos

**Figure 4:**
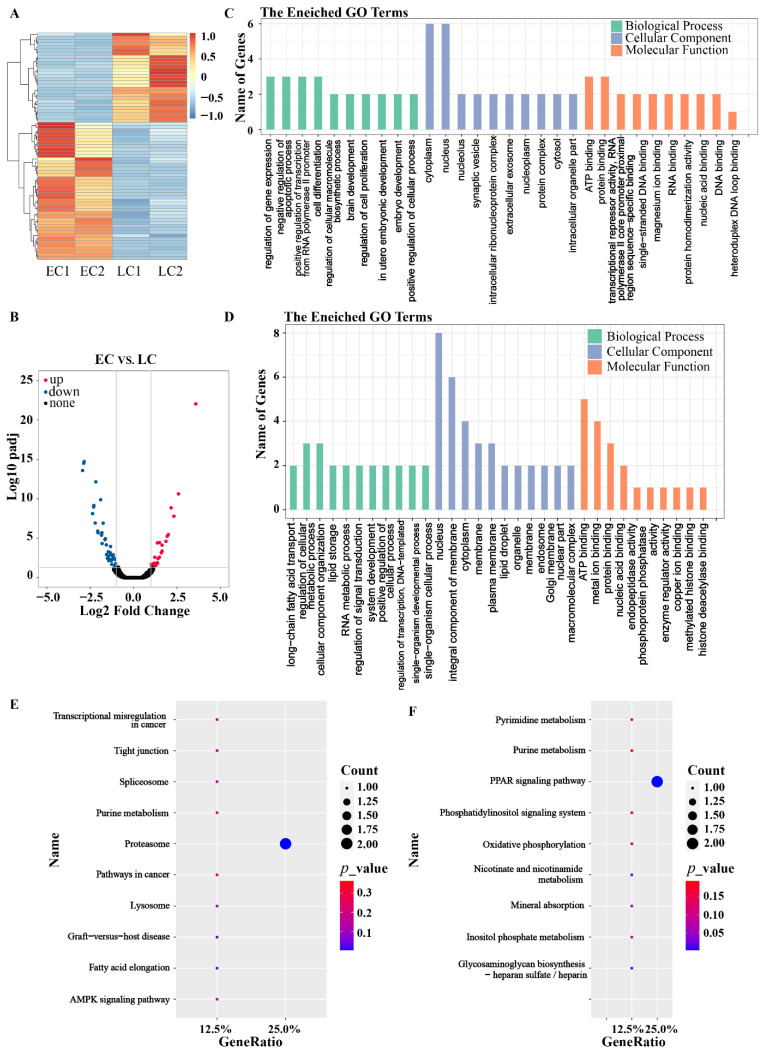
DEGs analysis between pig early cleavage embryos and late cleavage embryos of IVF with two repeats per group. (**A**). Volcano plot for DEGs between early and late cleavage pig IVF 2-cell embryos (|log_2_ FC| ≥ 1, FDR < 0.05). (**B**). Heatmaps of annotated DEGs in early and late cleavage pig IVF 2-cell embryos. GO enrichment analysis of (**C**) upregulated and (**D**) downregulated DEGs. KEGG pathway enrichment analysis of (**E**) upregulated and (**F**) downregulated DEGs.

For IVF embryos, there were 71 DEGs (|log_2_FC| ≥ 1, FDR < 0.05) between the EC and the LC embryos, including 43 significantly upregulated and 28 downregulated genes (Figure 4A,B). GO analysis showed that the DEGs were mainly enriched in gene expression and transcription-related terms, which showed positive regulation of cellular processes and negative regulation of apoptotic processes (Figure 4C,D). KEGG analysis presented that the DEGs were enriched in the proteasome, lysosome, and in the oxidative phosphorylation and PPAR signaling pathways (Figure 4E,F). Among them, the proteasome is one of the main degradation pathways of misfolded proteins and other useless cellular proteins during protein synthesis, which may be one of the key reasons caused embryonic retardation.

### 3.5. Quantitative Real-Time PCR Analysis of Genes Related to Apoptosis and Pluripotent in PA and IVF Embryos

**Figure 5:**
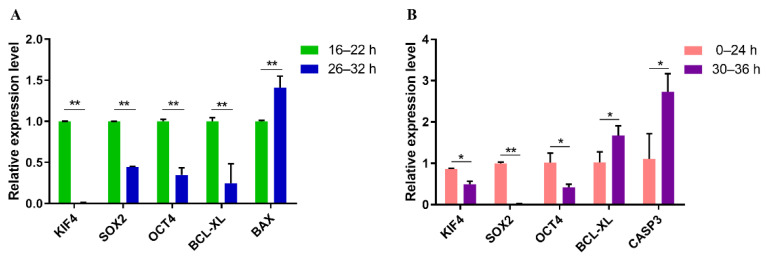
The relative mRNA abundance of some important genes. (**A**) Relative expression levels of pluripotency- and apoptosis-related genes of blastocysts in early and late cleavage PA embryos. (**B**) The relative expression levels of pluripotent and apoptosis-related genes in early and late cleavage IVF 2-cell embryos. *: *p* < 0.05; **: *p* < 0.01.

To verify the molecular mechanism underlying embryo development, the expression of genes related to apoptosis and pluripotency were analyzed using qRT-PCR. Results showed that the expression level of *OCT4*, *SOX2*, and *KIF4* in EC embryos was significantly higher than that in the LC group (Figure 5A,B). The expression of *BAX* in the PA blastocysts developed from EC embryos was significantly lower (*p* < 0.05) than that from LC embryos. However, in the PA blastocysts, the expression of *BCL-XL* in EC embryos is significantly higher than in LC embryos, which is opposite to the IVF 2-cell embryos (Figure 5B). The expression level of *CASP3* in the IVF EC embryos was found significantly lower than IVF LC embryos. These results support the analysis of RNA-seq.

### 3.6. Molecular Mechanism Underlying the Retardation of Development in Late Cleavage Embryos

The molecular mechanism underlying the better developmental potential of early cleavage embryos is summarized in Figure 3. The genes related to NER, including *DDB1*, *DDB2*, *ERCC5*, and *GTF2H4*, were significantly upregulated in late cleavage embryos, suggesting more severe DNA damage in the LC embryos. DNA damage triggers a series of signaling cascades promoting cellular survival, including DNA repair, cell cycle arrest, autophagy, and apoptosis. The *EDEM3*, *EIF2AK3*, and *PMAIP1*, which are related to the endoplasmic reticulum (ER) and apoptosis, were found overexpressed in late cleavage embryos, suggesting that the DNA damage further caused the ER stress. While the expression of cell cycle-related genes, such as *TFDP1*, *CDKN2*, and *CCCND3*, was found lower in late cleavage embryos, suggesting that the development of embryos was arrested until the completion of DNA repair. DNA damage also may cause a large number of misfolded proteins in the embryo, activate the p53 signaling pathway, and cause cell apoptosis. Higher expression of genes concerning the proteasome, lysosome, oxidative phosphorylation, and the PPAR signaling pathways, such as *PSMF1*, *CLN1*, *COX11*, and *PLIN2*, were found in the LC embryos. Moreover, the expression of genes related to P53 and the MTOR signaling pathway, including *RRAGB*, *mTOR*, *AMPK*, *EIF4EBP1*, *LPAR4*, *GNB1*, and *P21*, were also found significantly higher in late cleavage embryos, which supports the above opinion. Therefore, we hypothesize that the endoplasmic reticulum stress and DNA damage led to embryonic retardation in the LC embryos.

## 4. Discussion

The present study has proved that embryos with an early cleavage had a higher blastocyst development rate. However, the changes in gene expression between early cleavage and late cleavage porcine 2-cell embryos of PA and IVF were not investigated. Considering that Smart-seq2 is widely used in embryos for the analysis of gene expression, early cleavage and late cleavage porcine 2-cell embryos from PA and IVF were collected and sequenced in this study to investigate the molecular mechanism underlying the retardation of development in late cleavage embryos.

Selection of optimal embryos noninvasively before implantation is a key part of the study and application of in vitro cultured embryos, and the embryo scoring system has been the most widely used method. Embryo scoring systems, which are based on the morphology (pronuclear morphology, cell number, fragmentation degree, size and shape of blastomeres, multinucleation, and blastocyst characteristics [2,4]), are closely linked to embryo viability [26]. Pronuclear morphology evaluation with subsequent evaluation of embryo morphology significantly increases implantation rates [27]. However, the morphological evaluation is more like a subjective process due to drift in scoring, observer variability, and lack of definitive ways to assess the specific characteristics [28,29,30] and may not accurately reflect the developmental potential of embryos [3,4,5]. Moreover, time-lapse microscopy (TLM), which can track and collect visual information of individual embryos continuously without any disturbance, is a novel instrument for evaluating embryo viability [31]. However, TLM is not widely used because of its high cost. Using this instrument, studies found that the embryo development potential was related to the cleavage of early embryos and can be predicted in the early stage [32,33], which further supports the opinion that the selection of high-quality embryos based on the timing of first embryonic cleavage. The time of the first division of embryos can be used as one of the important bases for the early screening of high-quality embryos [34]. This method, based on first cleavage time, meets the minimal requirements of embryo selection, including standardization, ease of assessment, objectivity, minimal harm to the embryo, and a high correlation with pregnancy rates.

Abundant studies showed that early cleavage embryos are more likely to develop to the blastocyst stage in vitro [12]. The present study suggested similar results as previous studies that early cleavage embryos have better developmental potential following indicators, such as the cleavage rate, the blastocyst rate, and nucleus number in blastocysts. Although the cleavage rates of IVF embryos with early cleavage and late cleavage were not significantly different, the cleavage rate of early cleavage was higher than that of late cleavage in the data. It, to a certain extent, indicated that early cleavage embryos have a higher implantation potential. Evidence about the cell number of blastocysts of PA has suggested that early cleavage embryos also had better blastocyst quality. Unfortunately, blastocyst cell numbers from IVF embryos were not assessed due to the absence of materials.

As stated in the Materials and Methods section, in this study, embryos were observed 12–48 h after activation and fertilization. We decided that too-early-cleavage embryos were defined as those that undergo the first cleavage reaching the two-cell embryo stage by 16 h after PA, because the cleavage number, as well as the cleavage rate, was at a low level even though the blastocyst rate was considerable (Table 1). Similar results can be achieved with IVF embryos (Table 3). According to previous studies, faster development rates in vitro might be correlated with loss and aberrant expression of genes [9]. The general rule and molecular mechanisms of delayed cleavage need to be tracked and focused on, so embryos cleaving too early were discarded.

Early preimplantation embryos are precious and scarce samples that contain limited numbers of cells, which can be problematic for quantitative gene expression analyses. This material lack of barriers can be overcome by Smart-seq2 of scRNA-seq. In this study, significant changes in gene expression between early cleavage and late cleavage embryos of PA were observed. The activation of nucleotide excision repair, apoptosis, and autophagy suggested that DNA damage might happen in late cleavage embryos. On the other hand, the high expression of endoplasmic reticulum stress-related genes predicted misfolding in protein production in late cleavage embryos. It is uncertain whether DNA damage causes protein misfolding in the ER. It has been reported that 1896 DEGs were identified between SCNT early cleavage embryos and late cleavage embryos [19]. Similar results to ours showed that the DEGs were enriched in the ribosome, mRNA surveillance, protein processing in the endoplasmic reticulum, and spliceosome pathways [19].

Similar results were found in PA embryos. Spliceosome, lysosome, oxidative phosphorylation, and AMPK signaling pathways were also enriched in IVF embryos. Combined with the transcriptome of PA and IVF, the top KEGG pathway was nucleotide excision repair (NER), including *DDB1*, *DDB2*, *ERCC5*, and *GTF2H4* upregulated in late cleavage embryos. Both *DDB1* and *DDB2* expressed highly in late cleavage embryos recognize DNA damage. Then, *ERCC5* makes the 3′ incision, and *GTF2H4* acts by opening DNA around the lesion to allow the excision of the damaged oligonucleotide and its replacement by a new DNA fragment. The DEGs were also enriched in apoptosis, autophagy, the cell cycle, phagosome, and lysosome, which can be activated by DNA damage. The particular phosphorylation catalyzed by *STK4* (Serine/Threonine Kinase 4) is associated with apoptosis [35]. Nucleoplasmin-2 (NPM2) is a key gene in chromatin reprogramming, especially during fertilization and early embryonic development [36]. The effects of miR-191 on cell cycle arrest and cell apoptosis are abrogated by *YBX3* overexpression [37]. Additionally, the expression level of *KAZN* (Kazrin, Periplakin Interacting Protein), *MRPS26* (Mitochondrial Ribosomal Protein S26), *FOXO1* (Fork-head Box O1), and *TP44* (T-cell-specific Surface Glycoprotein) were significantly increased in our study. The abnormal activation of these genes may hinder the development of embryos. *TMEM59*, a type I transmembrane protein, and its homolog, *TMEM59L*, are localized in Golgi and endosomes [38]. Overexpression of them induces intrinsic caspase-dependent apoptosis. SET plays an inhibitory role in DNA damage caused by Granzyme A [39]. *NCAPD3* is essential for the assembly of metaphase chromosomes in HeLa cells [40]. These genes are involved in the regulation of the cell cycle and cell apoptosis, which may influence the cleavage of embryos. In addition, the expression of *GLCE*, *STEAP1*, *PGRMC2*, *ANKRA2*, *PLIN2*, and *NME7* were significantly decreased in the late cleavage embryos compared to the early cleavage embryos, which may be a disadvantage to the development of embryos. These genes are mainly enriched in pathways related to the proteasome, DNA repair, cell cycle arrest, autophagy, and apoptosis, suggesting that endoplasmic reticulum stress and DNA damage may be the major reasons for embryonic retardation and lower potential in LC embryos.

## 5. Conclusions

Our study showed porcine embryos that complete the first embryonic cleavage earlier showed better ability in the following development. Smart-seq2 suggested that early cleavage and late cleavage embryos had distinct RNA profiles and revealed a molecular mechanism underlying the retardation of development in late cleavage embryos. Based on the GO analysis and KEGG pathway database, we speculate that the severe endoplasmic reticulum stress and DNA damage led to embryonic retardation in LC embryos. This study provides new insights for following applications and offers important information to better understand the molecular mechanisms of the timely manner of porcine embryo development.

## Figures and Tables

**Table 1:** The effect of cleavage time on statistics of cleavage time of PA embryos.

Cleavage Time	Cleavage Number	Blastocyst Number	Cleavage Rate (%) *	Blastocyst Rate (%) *
0–16 h	2.75 ± 0.25	1.75 ± 0.25	1.82 ± 0.23	62.50 ± 4.17
16–18 h	7.00 ± 1.35	5.75 ± 1.31	4.42 ± 0.77	79.52 ± 5.30
18–20 h	22.50 ± 5.52	10.50 ± 2.47	14.58 ± 3.93	49.30 ± 6.02
20–22 h	48.25 ± 9.76	23.00 ± 4.36	29.95 ± 2.15	48.20 ± 1.39
22–24 h	32.50 ± 8.27	12.00 ± 2.94	20.02 ± 2.88	37.15 ± 1.53
24–26 h	13.00 ± 4.24	5.50 ± 1.85	7.50 ± 1.43	43.31 ± 6.46
26–28 h	8.50 ± 2.02	3.25 ± 1.11	5.14 ± 0.44	35.83 ± 6.29
28–30 h	6.50 ± 1.85	1.75 ± 0.48	4.04 ± 0.68	27.50 ± 4.33
30–32 h	4.00 ± 0.00	0.50 ± 0.29	2.74 ± 0.47	12.50 ± 7.22
32–42 h	10.50 ± 0.87	1.00 ± 0.00	7.37 ± 1.77	9.70 ± 0.72
42–48 h	3.75 ± 0.48	0.00 ± 0.00	2.43 ± 0.19	0.00 ± 0.00

* Cleavage rate refers to the proportion of cleavage embryos in a certain time to the total cleavage embryos. * Blastocyst rate refers to the percentage of cleaved embryos that developed into blastocysts; the value is represented as mean ± SEM.

**Table 2:** Development efficiency of early and late cleavage of porcine PA and IVF embryos.

Content	PA		IVF	
	EC	LC	EC	LC
Number of cleavage embryos	77.8 ± 14.0 ^a^	19.0 ± 3.5 ^b^	20.3 ± 3.5	11.3 ± 0.9
Cleavage rate (%)	48.9 ± 4.2 ^a^	11.9 ± 0.7 ^b^	41.7 ± 4.2	28.6 ± 4.7
Number of blastocysts	39.3 ± 6.0 ^a^	5.5 ± 1.8 ^b^	5.3 ± 1.9	1.7 ± 0.7
Blastocyst rate (%)	51.2 ± 2.2 ^a^	27.2 ± 6.0 ^b^	28.9 ± 3.9 ^a^	10.7 ± 1.9 ^b^
Primary blastocyst cell number	44.5 ± 4.2 ^a^	33.5 ± 2.7 ^b^		
Initial blastocyst cell number	49.7 ± 4.1 ^a^	40.3 ± 3.3 ^b^		
Expanded blastocyst cell number	74.8 ± 6.4 ^a^	50.7 ± 3.7 ^b^		
Hatching blastocyst cell number	65.0 ± 5.6	—		

^a, b^ Values with different superscripts within the row indicate a significant difference (*p* < 0.05). The value is represented as mean ± SEM. “—” indicates not detected.

**Table 3:** Statistics of cleavage time for IVF embryos.

Cleavage Time	Cleavage Number	Blastocyst Number	Cleavage Rate (%)	Blastocyst Rate (%)
0–18 h	2.00 ± 0.71	0.50 ± 0.29	3.85 ± 1.61	20.83 ± 12.50
18–20 h	2.25 ± 1.31	0.50 ± 0.29	4.94 ± 2.45	16.67 ± 11.79
20–22 h	5.50 ± 1.26	2.00 ± 1.08	11.35 ± 2.46	32.22 ± 11.71
22–24 h	8.75 ± 1.84	2.00 ± 0.41	17.54 ± 3.31	23.98 ± 3.71
24–26 h	6.00 ± 1.58	1.00 ± 0.41	11.33 ± 1.20	19.58 ± 7.08
26–28 h	5.75 ± 1.93	0.75 ± 0.48	10.34 ± 1.60	14.58 ± 8.59
28–30 h	5.75 ± 2.10	0.75 ± 0.25	10.67 ± 1.85	14.58 ± 6.25
30–32 h	4.25 ± 0.75	1.25 ± 0.48	8.92 ± 1.98	18.33 ± 10.67
32–42 h	5.50 ± 0.87	0.75 ± 0.48	11.94 ± 2.84	15.48 ± 5.87
42–48 h	5.75 ± 3.22	0.25 ± 0.25	9.13 ± 3.77	6.25 ± 6.25

## Data Availability

The scRNA-seq profiles of IVF embryos reported in this paper are available from NCBI Sequence Read Archive (accession number PRJNA758601). The scRNA-seq profiles of PA embryos are available from NCBI Sequence Read Archive (accession number PRJNA759843). The dates generated and analyzed during this study are included in this paper. Additional datasets used and/or analyzed during the current study are available from the corresponding author on reasonable request.

## Supplementary Materials

The following supporting information can be downloaded at: https://www.mdpi.com/article/10.3390/genes13071251/s1, Table S1: The primers used in PA embryos for qRT-PCR; Table S2: The primers used in IVF blastocysts for qRT-PCR; Table S3: GO terms significantly (q-value < 0.05) enriched from DEGs between early and late cleavage embryos of PA.
