# Supplementary material for: RNA-Seq Reveals the Underlying Molecular Mechanism of First Cleavage Time Affecting Porcine Embryo Development
Source: Genes (Basel). 2022 Jul 15;13(7):1251. doi: 10.3390/genes13071251 (PMC9320770; doi:10.3390/genes13071251)
Supplement: Supplementary file 1 [file genes-13-01251-s001.zip › genes-1788681-supplementary.pdf]

Supplementary Materials:

Table S1. The primers used in PA embryos for qRT-PCR

Name of primer	Gene symbol	Sequence of primer (5′-3′)	Tm (℃)	Fragment size (bp)
18*S -F*<br>18S *-R*	18S ribosomal RNA	GGACCTGACCGACTACCTCA<br>CCATCTCCTGCTCGAAGTCC	58	134
SOX2 -F<br>*SOX2 -R*	SRY-Box Transcription Factor 2	ACCAGAAGAACAGCCCAG<br>CCGTCTCCGACAAAAGTT	55	159
OCT4 -F<br>*OCT4 -R*	POU Class 5 Homeobox 1	GTGTTCAGCCAAACGACCAT<br>TTGCCTCTCACTCGGTTCTC	55	200
KLF4 -F<br>*KLF4 -R*	Kruppel Like Factor 4	TCCCACCGCTCCATTAC<br>ATGAGAACTCTTCGTGTAGG	55	162
BCL-XL -F<br>*BCL-XL -R*	BCL2 Like 1	ACTGAATCAGAAGCGGAAAC<br>AAAGCTCTGATACGCTGTCC	60	249
BAX -F<br>*BAX -R*	BCL2 Associated X, Apoptosis Regulator	CTAGAACCTAGCAGCACCAT<br>CGATCTTGGTGAAGTACTC	60	151

Table S2. The primers used in IVF blastocysts for qRT-PCR

Name of primer	Gene symbol	Sequence of primer (5′-3′)	Tm (℃)	Fragment size (bp)
18S -F<br>*18S -R*	18S ribosomal RNA	GGACCTGACCGACTACCTCA<br>CCATCTCCTGCTCGAAGTCC	58	134
SOX2 -F<br>*SOX2 -R*	SRY-Box Transcription Factor 2	TCCATGACCAGCTCGCAGACC<br>TCGCCTCGGACTTGACCACT	55	121
OCT4 -F<br>*OCT4 -R*	POU Class 5 Homeobox 1	GTGTTCAGCCAAACGACCAT<br>TTGCCTCTCACTCGGTTCTC	55	200
KLF4 -F<br>*KLF4 -R*	Kruppel Like Factor 4	GACCTACTTACTCGCCTTGCT<br>CCCGAACCCCAGTCAACGAA	55	199
BCL-XL -F<br>*BCL-XL -R*	BCL2 Like 1	ACTGAATCAGAAGCGGAAAC<br>AAAGCTCTGATACGCTGTCC	60	249
CASP3 -F<br>*CASP3 -R*	Caspase 3	GGCGTGTCAGAAAATACCAG<br>TTAACCCGAGTAAGAATGTG	55	174

Table S3. GO terms significantly (q-value < 0.05) enriched from DEGs between early and late cleavage embryos of PA.

GO Terms	GO Branch	Term Name	p-value	q-value
GO:0003676	molecular function	nucleic acid binding	2.74612E-05	0.027324

GO:0044424	cellular components	intracellular part	1.06632E-10	6.86E-08
GO:0043226	cellular components	organelle	2.69831E-05	0.002892
GO:0043229	cellular components	intracellular organelle	2.18552E-06	0.000468
GO:0043227	cellular components	membrane-bounded organelle	2.47549E-05	0.002892
GO:0043231	cellular components	intracellular membrane-bounded organelle	1.85939E-06	0.000468
GO:0005634	cellular components	nucleus	2.56679E-05	0.002892
